# Risk factors spatial-temporal detection for dengue fever in Guangzhou

**DOI:** 10.1017/S0950268818002820

**Published:** 2018-10-26

**Authors:** Lingcai Kong, Chengdong Xu, Pengfei Mu, Jialiang Li, Senyue Qiu, Haixia Wu

**Affiliations:** 1 Department of Mathematics and Physics, North China Electric Power University, Baoding, China; 2 State Key Laboratory of Resources and Environmental Information System, Institute of Geographic Sciences and Natural Resources Research, Chinese Academy of Sciences, Beijing, China; 3 Electrical & Electronic Engineering School, North China Electric Power University, Baoding, China; 4 State Key Laboratory of Infectious Disease Prevention and Control, Collaborative Innovation Center for Diagnosis and Treatment of Infectious Diseases, National Institute for Communicable Disease Control and Prevention, Chinese Center for Disease Control and Prevention, Beijing, China

**Keywords:** Dengue fever, determinant power, GeoDetector, risk factors

## Abstract

Dengue fever (DF) has been a growing public-health concern in China since its emergence in Guangdong Province in 1978. Of all the regions that have experienced dengue outbreaks in mainland China, the city of Guangzhou is the most affected. This study aims to investigate the potential risk factors for dengue virus (DENV) transmission in Guangzhou, China, from 2006 to 2014. The impact of risk factors on DENV transmission was qualified by the *q*-values calculated using a novel spatial-temporal method, the GeoDetector model. Both climatic and socioeconomic factors were considered. The impacts on DF incidence of each single factor and the interaction of two factors were analysed. The results show that the number of days with rainfall of the month before last has the highest determinant power, with a *q*-value of 0.898 (*P* < 0.01); the *q*-values of the other factors related to temperature and precipitation were around 0.38–0.50. Integrating a Pearson correlation analysis, nonlinear associations were found between the DF incidence in Guangzhou and the climatic factors considered. The coupled impact of the different variables considered was enhanced compared with their individual effects. In addition, an increased number of tourists in the city were associated with a high incidence of DF. This study demonstrates that the number of rain days in a month has great influence on the DF incidence of the month after next; the temperature and precipitation have nonlinear impacts on the DF incidence in Guangzhou; both the domestic and overseas tourists coming to the city increase the risk of DENV transmission. These findings are useful in the risk assessment of DENV transmission, to predict DF outbreaks and to implement preventive DF reduction strategies.

## Introduction

Dengue fever (DF) is a mosquito-borne viral infection caused by any one of four serotypes of dengue virus (DENV 1–4) [1]. It is a major public-health concern throughout tropical and sub-tropical regions. In recent decades, the incidence of DF has grown dramatically worldwide, making it the most important mosquito-borne viral disease [2, 3]. It has been estimated that 390 million dengue infections occur per year, of which 96 million manifest clinically (with any severity of disease) [3]. Brady *et al*. have estimated that more than 3.9 billion people in 128 countries are at risk of infection with dengue viruses [4]. The most seriously affected regions include Africa, the Americas, the Eastern Mediterranean, South-East Asia and the Western Pacific [3–5]. The drivers of the rapid dengue expansion include urbanisation, globalisation (travel and trade), the lack of effective mosquito control and climate change [6, 7].

The first outbreak of DF in mainland China occurred in 1978 in the city of Foshan in Guangdong Province. Since then, DF outbreaks have been recorded sequentially in Hainan, Guangdong, Guangxi, Fujian, Zhejiang and Yunnan provinces [8]; the highest number of DF cases reported in China occurred in Guangdong Province [9]. In 2014, the most severe DF outbreak in history occurred in Guangdong Province, with more than 45 000 cases of infection [8–10]. In this outbreak, 37 359 cases of infection were reported in Guangzhou, the provincial capital of Guangdong Province, seven times the historical record, confirming Guangzhou as the most affected city [11]. DF outbreaks in China were previously thought to be imported [9], but recent studies suggest that DF may be endemic to China [12, 13].

DENV are transmitted by *Aedes* mosquitoes, which are highly sensitive to climate, as temperature, precipitation and humidity, for example, can influence dengue transmission both directly and indirectly mediated by mosquito density [14, 15]. The effects of climatic factors on DF incidence at different times and in different regions have been studied [16–20] and can vary dramatically according to region [15, 21]. Many studies have investigated the relationship between DF incidence and climatic factors in Guangzhou, most have employed regression models, such as the Poisson model [22], the negative binomial model [23], the zero-inflated regression model [24] and the distributed lag non-linear models [25]. Although there are some inconsistencies, the results of these studies have provided valuable implications for dengue risk assessment, prediction and prevention. However, the relationship between climate factors and dengue transmission is complex [15]; therefore, further research is needed.

Socioeconomic factors, such as urbanisation, also influence the incidence of DF [15, 18, 26]. One study investigated the effects of socioeconomic and environmental factors on the incidence DF at the township level in the Pearl River Delta economic zone in 2013 [27]. Another study examined the effects of socioecological factors on the spatial distribution of DF in the unprecedented 2014 outbreak in Guangzhou [28]. Both these studies employed annual data. However, some socioeconomic factors, such as the number of tourists, may vary dramatically in a year. To our knowledge, no study on the effects of such factors on the incidence of DF has been published.

In this study, we use a novel spatial-temporal method, the GeoDetector, to identify the potential climatic and socioeconomic factors associated with the DF incidence in Guangzhou. The results may be useful in understanding the relationship between DF incidence and risk factors in Guangzhou, in providing information to predict DF outbreaks, and in developing preventive measures.

## Methods

### Study area

**Fig. 1.**
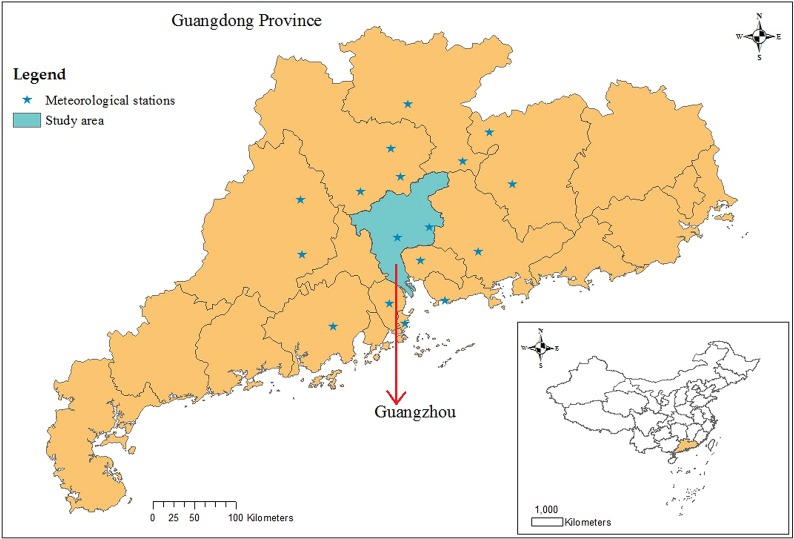
Geographic location of Guangdong Province and Guangzhou city in China, and meteorological stations used to interpolate the meteorological data in Guangzhou.

Guangzhou, the city most affected by dengue in mainland China, is located on the south-east coast of China (22°26′–23°56′N, 112°57′–114°3′E, Fig. 1) and had a population of 13.08 million in 2014 [29]. As the capital of Guangdong Province, Guangzhou serves as the political, economic, scientific, educational, tourism and cultural centre in southern China. The city has a humid subtropical climate; summers are wet with high temperatures and high humidity, and winters are mild and comparatively dry.

### Materials

#### Guangzhou DF cases

**Fig. 2.**
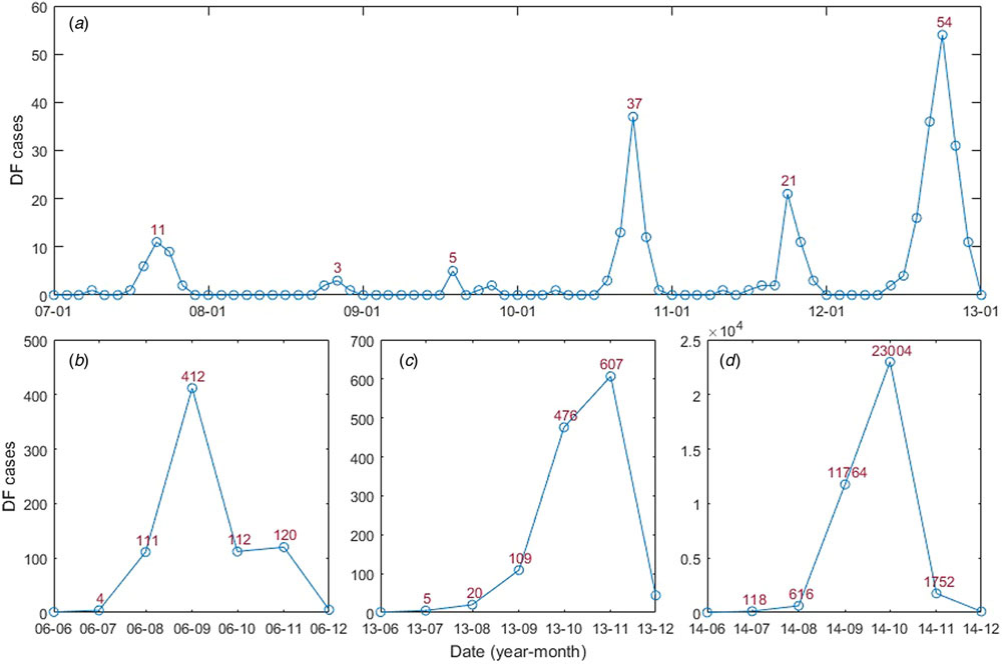
Guangzhou DF monthly cases from January 2006 to December 2014: (A) January 2007 to January 2013; (B) June 2006 to December 2006; (C) June 2013 to December 2013 and (D) June 2014 to December 2014.

In this study, monthly DF cases from January 2006 to December 2014 (Fig. 2) were obtained from the Chinese Center for Disease Control and Prevention (CDC, http://www.chinacdc.cn/). DF incidence was calculated by determining the ratio between the number of DF cases and the population size of the year. All four serotypes had been detected in Guangzhou, which was dominated by DENV 1–2; DENV 3 was first detected in 2009 and DENV 4 re-emerged in Guangzhou in 2010 [13, 30].

#### Potential risk factors

**Fig. 3.**
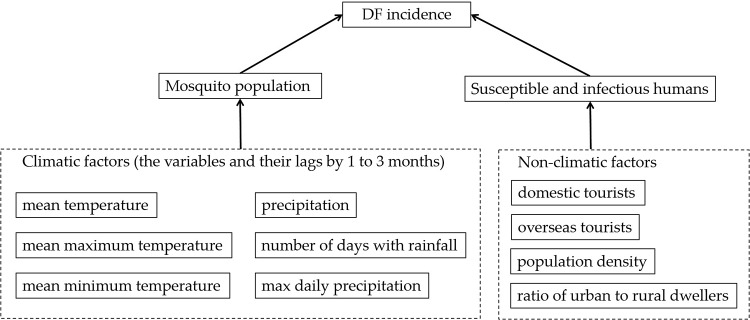
Potential risk factors to DF considered.

Transmission of DENV requires multiple factors: virus must be present (for example, imported cases), sufficient susceptible humans to the virus and contact between humans and mosquito vectors. While the first and second factors are usually related to socio-economic factors (for example, population density, travellers go or from abroad), the third factor is influenced by environmental factors, including climatic factors, living conditions, land cover type, etc. In this study, we examined the effects of both climatic and socioeconomic risk factors (Fig. 3) on the DF incidence in Guangzhou.

**Fig. 4.**
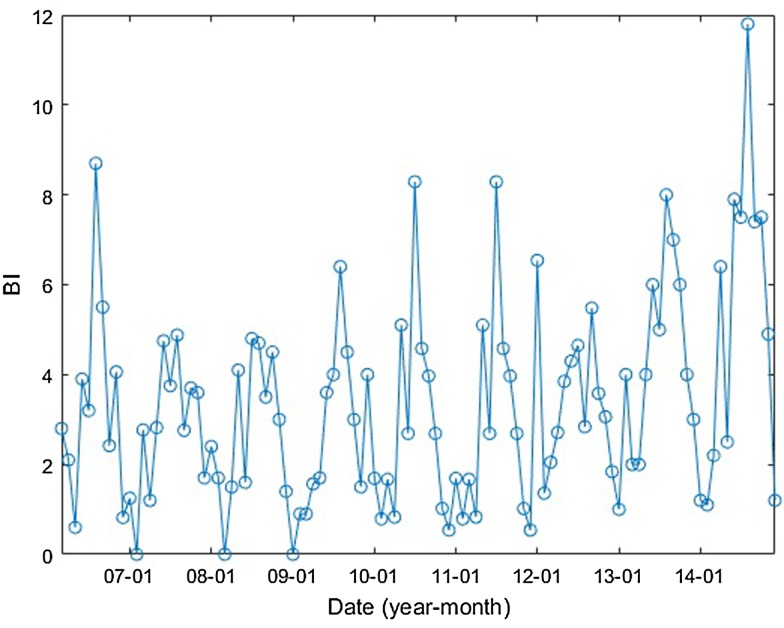
Guangzhou monthly BI between January 2006 and December 2014.

In Guangzhou, *Aedes albopictus* was the only vector species that transmitted DENV [9, 31]. The Breteau index (BI), calculated as the number of containers positive for *Aedes* mosquito larvae per 100 houses [1], was used to measure the mosquito population. The monthly BI of Guangzhou is shown in Figure 4.

**Fig. 5.**
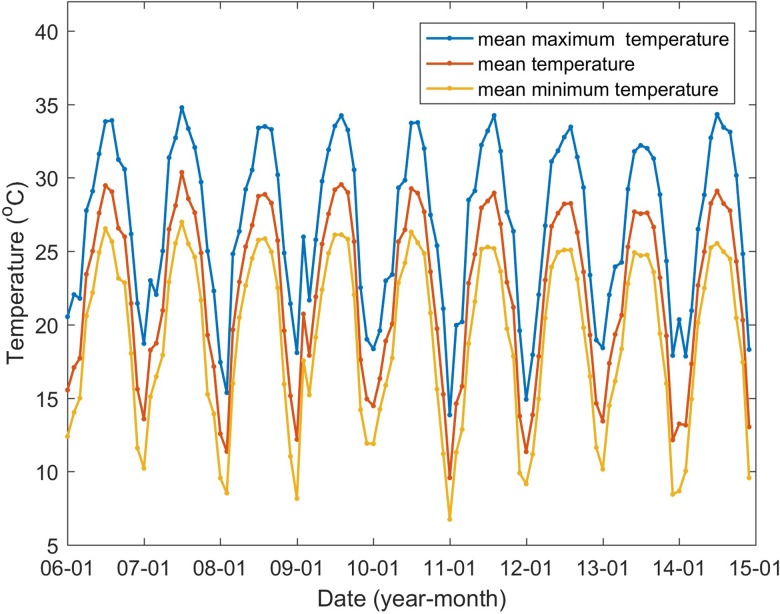
Guangzhou monthly mean, mean maximum and mean minimum temperatures between January 2006 and December 2014.

**Fig. 6.**
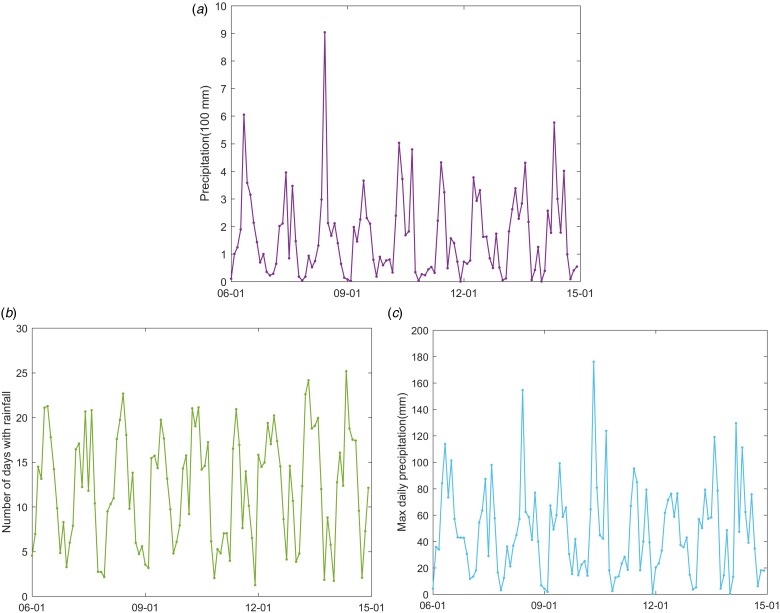
Monthly precipitation (A), number of days with rainfall (B) and maximum daily precipitation (C) in Guangzhou between January 2006 and December 2014.

The climatic variables influencing the mosquito population considered include the monthly mean temperature (*T*), mean maximum temperature (*T*_max_), mean minimum temperature (*T*_min_), precipitation (*P*), the number of days with rainfall (*R*) and maximum daily precipitation (*P*_max_), as well as their lags of 1–3 months (*V*^−*j*^, *j* = 1,  2,  3, *V* denotes one of the former symbols). These data from meteorological stations located in Guangzhou and within 100 km of the borders of Guangzhou (Fig. 1) between 2006 and 2014 were obtained from the China Meteorological Data Sharing Service System (http://data.cma.cn/). We then employed the inverse distance weighted interpolation method to obtain the corresponding data for Guangzhou. The monthly mean, mean maximum and mean minimum temperatures are shown in Figure 5, the other climatic factors are shown in Figure 6.

**Fig. 7.**
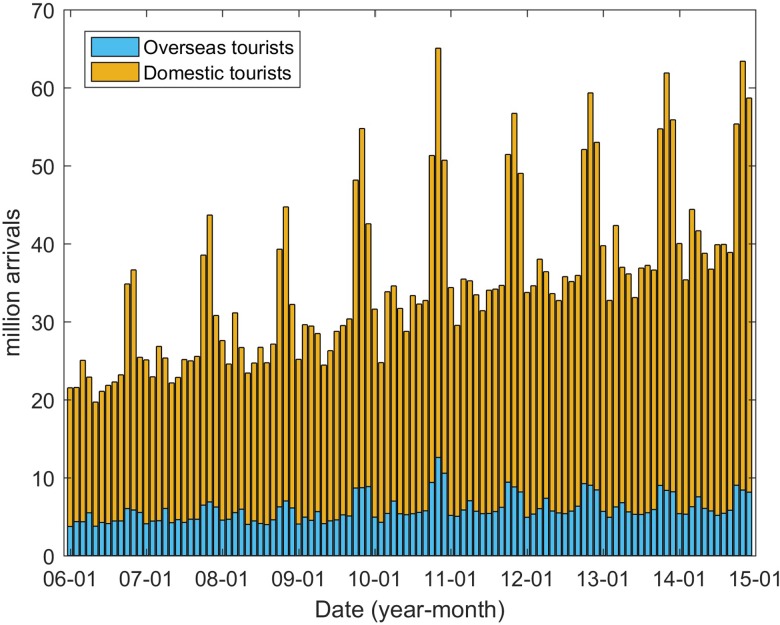
Tourists coming to Guangzhou between January 2006 and December 2014.

In addition to the climatic factors, socioeconomic factors were also considered to be potentially important drivers in DENV transmission [18, 26]. In this study, we collected the data pertaining to monthly domestic (*D*) and overseas tourists (*O*) coming to Guangzhou from the Guangzhou Statistics Bureau (http://www.gzstats.gov.cn/). Figure 7 shows the variations in the number of tourists over time. Population density and the proportion of urban and rural dwellers were also considered potential risk factors to DENV transmission.

#### GeoDetector

Several studies have reported the associations between DF incidence in Guangzhou and risk factors [22–25]. However, these methods usually limited to some shortcomings, for example, the relationship between DF incidence and risk factors are usually nonlinear; the risk factors may have an interactive effect on DF incidence but cannot be fully reflected by regression models. The above limitations do not exist in the case of the GeoDetector model [32], of which, the basic idea is to measure the correspondence of the spatial temporal distribution of response variables (e.g. DF incidence) to that of suspected determinants (e.g. climatic factors). It assumes that if the suspected determinant is a disease risk, the spatial temporal distribution of the disease should be similar to that of the factor. That is to say, if a potential factor (*X*) causes a disease (*Y*), their temporal-spatial distributions tend to be consistent [32], as measured by the power of determinant:
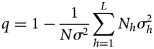
where *σ*^2^ denotes the variance of *Y* in the study area; *N* is the size of the population *Y*, which is composed of *L* strata (*h* = 1, 2, …, *L*) and 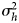 stands for the variance within stratum *h*. The value of the *q*-statistic, ranging from 0 to 1, denotes the determinant power of a potential risk factor *X*. This means that the factor explains 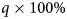 of the DF incidence. The bigger it is, the more determinant power of the factor *X*. If *q* = 1, *Y* is determined by *X* completely. On the contrary, if *q* = 0, the factor *X* is unrelated to *Y* completely. In addition to extracting the determinant power of a single factor, the *q*-statistic can also be used to probe the determinant power of the interactive effect of two risk factors, *X*_1_ and *X*_2_. By comparing the interactive effect between two factors with the effect of their individual contributions to *Y*, the interactive effects are determined (shown in Table 1). Detailed information about the GeoDetector model can be found in [32–34].

**Table 1.** Types of interaction between two factors

Interactive effects	Condition
Weaken	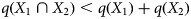
Weaken, univariate	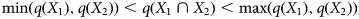
Weaken, nonlinear	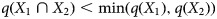
Enhance, bivariate	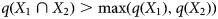
Enhance, nonlinear	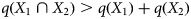
Independent	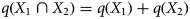

The symbol of “
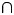
” means the interaction of two factors, and 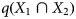 represents the *q*-value for the interaction between two factors *X*_1_ and *X*_2_.

The GeoDetector model can handle both quantitative and nominal data, and thus offer a novel approach for detecting interactions between the risk factors for infectious diseases, such as bacillary dysentery [35] and hand-foot-and-mouth disease [36–38]. In this study, we used three geographical detectors, i.e. factor detector, ecological detector and interaction detector to identify, respectively, the responsible factors, the relative importance factors and the interaction relationship for each two factors. The method was implemented using software downloaded from the GeoDetector website at http://www.geodetector.org/. All variables are temporal variables but without spatial variation. The population density and the proportion of urban and rural dwellers were stable in time within a year, while the climatic variables and tourists coming to Guangzhou varied monthly. To use the model, the risk factors measured by continuous data need to be discretised. The most commonly used discretisation methods include the equal interval method, the quantile method, the natural breaks method, the geometrical interval method, the standard deviation method, *etc*. [39]. Here, we used the *q*-statistic as the indicator for the assessment of the discretisation method and obtained the stratification information [39].

## Results

### Descriptive statistics

There were a total of 39 695 DF cases between January 2006 and December 2014. An obvious seasonal pattern in the number of DF cases can be seen. Most cases occurred in the July–November period, peaking in September and October (Fig. 2). The DF incidence also shows striking annual variations, three large outbreaks occurred in 2006, 2013 and 2014, with the highest number of cases recorded in 2014 (37 359 cases). The DF cases and the climatic and socioeconomic factors have been summarised (Table 2).

**Table 2.** Statistical description of incidence and potential risk factors of DF

Variables	Mean ± s.d.	Quantiles				
		5%	25%	50%	75%	95%
DF cases	367.55 ± 2478.47	0	0	1	7.5	453.6
BI	3.36 ± 2.26	0.67	1.62	2.92	4.58	7.80
Mean temperature (°C)	22.15 ± 5.69	12.74	17.55	23.12	27.59	29.08
Mean max temperature (°C)	26.78 ± 5.63	17.92	22.04	27.73	31.86	33.81
Mean min temperature (°C)	19.04 ± 5.77	9.30	14.82	20.28	24.71	25.84
Precipitation (mm)	163.76 ± 155.92	5.36	47.29	127.86	225.80	431.34
Number of rainy days	12.08 ± 6.24	2.35	6.41	12.35	17.25	21.11
Max daily precipitation (mm)	47.1 ± 34.79	3.86	18.10	41.91	64.63	112.84
Ratio of urban to rural population	9.17 ± 0.44	8.68	8.80	9.08	9.51	10.07
Number of overseas tourists	59.37 ± 16.63	40.97	46.72	55.10	62.91	90.30
Number of domestic tourists	293.22 ± 91.77	177.99	222.18	280.87	332.15	478.24
Population density (person/km^2^)	1611.53 ± 148.2	1340.61	1500.24	1709.57	1726.96	1759.46

‘s.d.’ denotes the standard deviation of the corresponding variable.

### Results from the GeoDetector

We used the GeoDetector method to detect the determinant power of each risk factor and their interactive effects to the DF incidence in Guangzhou. The potential risk factors include six climatic factors (the monthly mean temperature, mean maximum temperature, mean minimum temperature, precipitation, number of days with rainfall and maximum daily precipitation) and four socioeconomic factors (the proportion of urban and rural dwellers, population density and overseas and domestic tourists). The determinant power of each factor and their interactive effects were qualified by the *q*-value calculated using the GeoDetector model. The association between the DF incidence and each factor – for example, a positive or negative relationship – was assessed using Pearson correlation coefficients.

The DF incidence demonstrates an apparent seasonal variation. This variation is correlated with the mosquito density and climatic factors and/or their lags of 1–3 months with different determinant powers. The *q*-values of BI and the climatic factors are shown in Table 3. The results show that the determinant power of BI is almost 60%, indicating a close relationship between BI and DF incidence. Among the climatic factors, the number of days with rainfall of the month before last has the highest determinant power, with a *q*-value of 0.898 (*P* < 0.01). However, the Pearson correlation was not significant (different from zero). We suppose that a nonlinear relationship exists between the DF incidence and the number of rainy days of the month before last. Regarding precipitation, the determinant power with the 2-month lag is the highest among the other lags, with a *q*-value of 0.486 (*P* < 0.01). The maximum daily precipitation in the last 2 months had the same *q*-values, 0.486. However, the Pearson correlation coefficients between DF incidence and them were not significant, meaning nonlinear relationships may exist between them. The interpretation for the nonlinear relationships can be found in the next section.

**Table 3.** *q*-values for mosquito density index and climatic factors and their lags of month

Factor	Months of lags			
	0	1	2	3
BI	0.595	–	–	–
Mean temperature	0.387	0.394	0.397	0.492
Mean max temperature	0.485	0.397	0.402	0.487
Mean min temperature	0.386	0.390	0.397	0.438
Precipitation	0.384	0.408	0.486	0.395
Number of rainy days	0.390	0.391	0.898	0.457
Max daily precipitation	0.384	0.486	0.486	0.387

*Note:* all the *q*-values are significant from zero with *P* < 0.05.

The determinant power of the mean temperature, mean maximum temperature, mean minimum temperature and their lags of 1–3 months are similar, with *q*-values within the range of 0.38–0.50 (*P* < 0.01). The Pearson correlations between these variables and the DF incidence were not significant (*P* > 0.05). This may be because the impact of these climatic variables and these lags on the DF incidence were nonlinear. The Pearson correlation coefficients between DF incidence and each risk variable considered are shown in Table 4.

**Table 4.** Pearson correlation coefficients between DF incidence and variables related to climate

Factor	Months of lags			
	0	1	2	3
Mean temperature	0.079^#^	0.146^#^	0.166*	0.171*
Mean max temperature	0.106^#^	0.166*	0.182*	0.181*
Mean min temperature	0.065^#^	0.139^#^	0.158^#^	0.163*
Precipitation	−0.111^#^	0.025	0.143^#^	0.074^#^
Number of rainy days	−0.166*	−0.006^#^	0.119^#^	0.145^#^
Max daily precipitation	−0.126^#^	−0.001^#^	0.070^#^	0.020

^#^*P* > 0.1; *0.05 < *P* < 0.1.

The determinant powers of socioeconomic factors were also calculated using the GeoDetector method. Both domestic and overseas tourists coming to Guangzhou have significant effects on DF incidence, with *q* values of 0.489 and 0.487, respectively (both *P* < 0.01). More tourists were associated with higher DF incidence. The Pearson correlation coefficients between domestic and overseas tourists and the DF incidence are 0.207 (*P* < 0.05) and 0.174 (0.05 <*P* < 0.1), respectively. For the population density and the proportion of urban and rural dwellers, neither has significant explanatory power, with *q*-values of 0.0723 (*P* = 0.1234) and 0.0671 (*P* = 0.1503).

From the former results, we found that, for each risk factor related to temperature, the factor with 3-months lag has the largest *q*-value; for the factors related to precipitation, is the one with 2-months lag. We analysed their interactive effects with BI, the domestic and overseas tourists. The results show that the coupled impact of every two factors considered was enhanced compared with the effect of their individual influences (Table 5). As we know, both higher temperature and more precipitation would provide more suitable conditions for mosquitoes, the results show that their interactive effect is enhanced. DF in Guangzhou was inspired by imported cases [30], travellers go to or from countries where DF is endemic have chance to import DENV. Coupled with greater mosquito population, or suitable environmental and climatic factors, more travellers would increase the risk of DF outbreak, caused enhanced interactive effects.

**Table 5.** The *q*-values for the interactive effect of different factors

Factors	BI	*T*^−3^	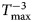	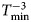	*P*^−2^	*R*^−2^	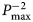	*D*	*O*
BI									
*T*^−3^	0.595^EB^								
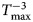	0.994^EB^	0.487^EB^							
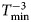	0.898^EB^	0.439^EB^	0.994^EN^						
*P*^−2^	0.994^EB^	0.591^EN^	0.994^EN^	0.994^EN^					
*R*^−2^	0.899^EB^	0.994^EB^	0.994^EB^	0.899^EB^	0.994^EB^				
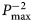	0.994^EB^	0.487^EB^	0.994^EN^	0.994^EN^	0.994^EN^	0.994^EN^			
*D*	0.995^EB^	0.489^EB^	0.995^EN^	0.995^EN^	0.996^EN^	0.996^EB^	0.996^EN^		
*O*	0.994^EB^	0.593^EN^	0.994^EN^	0.994^EN^	0.994^EN^	0.994^EB^	0.994^EN^	0.996^EN^	

EB, enhance (bivariate); EN, enhance (nonlinear).

## Discussion

DF has been a growing public-health concern in mainland China since its emergence in Guangdong Province in 1978 [40]. The city of Guangzhou, the provincial capital of Guangdong Province and one of the largest modern cities in China, has had the highest DF incidence in mainland China [11]. In this study, the determinant powers of potential climatic and socioeconomic factors were examined. The results indicate that the number of rainy days of the month before last had the greatest determinant power; the BI, the temperature and its lags of 1–3 months, precipitation, the maximum daily precipitation and the domestic and overseas tourists had significant impact on DF incidence in Guangzhou. The interactive effects of the mean minimum temperature and the other climatic variables were enhanced compared with the effect of their individual effects.

There is an obvious seasonal pattern in the Guangzhou DF incidence. This seasonal variation is associated with climatic factors through direct and indirect effects [14, 15]. There is an opposite effect of precipitation on mosquito populations. While precipitation provides habitats for the aquatic stages of the mosquito life cycle, and higher precipitation is associated with increased mosquito populations; intense rainfall may wash out breeding sites and therefore have a negative effect on mosquito populations [15]. This study shows that the number of days with rainfall of the month before last has great impact on the DF incidence, with a *q*-value of 0.898. This is consistent with the findings in [14], which show the number of days with rainfall to be a better predictor. The determinant powers of the other variables related to rainfall, the precipitation, the maximum daily precipitation and their lags of 1–3 months, were around 0.38 to 0.50. A non-significant positive association between DF outbreak risk and precipitation was detected in [14]. In this study, integrating the determinant powers calculated using the GeoDetector model with the Pearson correlation analysis, we found that nonlinear relationships exist between the DF incidence and the variables related to rainfall.

While precipitation plays an essential role in providing habitats for the aquatic stages of the mosquito, temperature has a direct effect on DENV replication within vectors and indirect effects on DENV transmission by influencing the mosquito development and survival [15]. This study also shows that temperature and its lags of 1–3 months are important factors that influence the DF incidence in Guangzhou. Higher temperature is associated with higher DF incidence, which is a positive association. Like precipitation, the relationships between DF incidence and the three variables related to temperature, as well as their lags of 1–3 months, may be nonlinear. It is known that mosquito population dynamics, the oviposition rates and transition and mortality in different stages of the life cycle are considerably influenced by temperature [15, 41–43]. In short, an ideal range for survival and development through all phases of the mosquito life cycle occurs, for example, at 20–30 °C, as stated in [44]. The development of mosquitoes is often slower in both cooler and higher temperatures [41, 42], although the critical limiting values may be different [15]. Temperature is also a key component in the ecology of DENV in that increasing temperature can accelerate viral replication within the vector and shorten the extrinsic incubation period (EIP) [45, 46]. Because of the variation in temperature throughout the day in nature, the effect of diurnal temperature ranges (DTRs) on the susceptibility of *Ae. aegypti* to DENV to DENV has also been explored in [47]. The authors found that compared with moderate DTRs or constant temperature, larger DTRs decreased the probability of infection of *Ae. aegypti*, but the EIP was unchanged [47]. Synthesising the above results, the overall effect of temperature on DENV ecology is complex; increasing temperature may accelerate parts of the viral transmission cycle, while other variables may become limited by higher temperatures [15]. Therefore, the nonlinear relationship between DF incidence and temperature is readily comprehensible.

DF incidence is also influenced by socioeconomic factors, such as population density, road density and land cover [27, 28, 48]. In this study, we examined the determinant power of domestic and overseas tourists coming to Guangzhou, the population density and the proportion of urban and rural dwellers. The results show that both domestic and overseas tourists coming to Guangzhou have a high determinant power of 0.489 and 0.487, respectively. There was a significant positive correlation between the tourists and DF incidence. The situation is similar to that in Australia, where there is an increasing trend in DF incidence as the number of overseas visitors increases [49]. Residents in Guangdong Province keep close connections with South East Asian countries that are in the DF-endemic regions [50]. Therefore, more travellers from these countries increase the risk of DENV transmission. We also explored the influence of population density and the proportion of urban and rural dwellers. The results show that these factors had non-significant determinant power in relation to DF incidence. This is inconsistent with the findings in [27], which analysed the relationships on a finer spatial scale. This inconsistency may be because the data we used were aggregated over large spatial scales.

The findings in this study have implication for the risk assessment, early warning of DF in Guangzhou, and to implement preventive strategies. Special attention should be paid to the rainy days, which has the largest determinant power on DF incidence. Besides mosquito density and climatic factors, which have been emphasised in the literatures [22–25], travellers to the city, especially to or from countries where DF is endemic, should also be noted.

Our study aims to detect the impact of climatic and socioeconomic factors on DF incidence in Guangzhou. However, there were some limitations. First, imported DF cases were not excluded due to a lack of information. Nevertheless, this problem has little influence on the results because the imported cases only accounted for <1% of all DF cases in Guangzhou in 2006–2014 [22, 25]. Second, we used aggregated data for the whole city, while the associations between DF and risk factors are site-specific [15, 21]. This may result in some detailed information been submerged; therefore, finer-scale research is needed in the future. Third, unreported and inapparent cases are an inherent limitation in the DF surveillance data. However, our results still have significance in the risk assessment of DENV transmission, in the prediction of DF outbreaks, and in implementing preventive DF reduction strategies.
